# A personal perspective on the nature of *npj Biofilms and Microbiomes*

**DOI:** 10.1038/npjbiofilms.2015.3

**Published:** 2015-03-25

**Authors:** Roberto Kolter

**Affiliations:** 1 Department of Microbiology and Immunobiology, Harvard Medical School, Boston, MA, USA

By now you, dear reader, will have had an opportunity to read our Editor-in-Chief Staffan Normark’s comments on the aims and scope of this new (and exciting!) journal ‘*npj Biofilms and Microbiomes*’.^1^ Let me at the very outset of these few lines emphatically echo Staffan’s opinions on the central importance of biofilms and microbiomes on the workings of our planet. The ubiquity of multispecies communities on Earth makes it imperative that they be carefully studied, often through the application of multidisciplinary approaches. This new journal clearly aims to serve as the home to the most exciting and innovative manuscripts in these areas. Backed by the outstanding publishing infrastructure and reputation of Nature Partner Journals, *npj Biofilms and Microbiomes* will in all likelihood get off to a fast start. At least, such is my hope. But do we really need another journal to publish work on biofilms and microbiomes? Do we not already have enough high quality journals that cover these areas of the microbial sciences? In the spirit of full disclosure, I did ask myself these—and many related questions—when Staffan initially approached me with the invitation to serve as Associate Editor. Obviously, in the end I agreed to serve as Associate Editor. Herein I would like to outline my reasons; these go way beyond the scientific aims and scope of the journal. Rather, I was most swayed by what I see as the need to revitalize the scientific publishing process. In the end, I felt convinced that the Editor-in-Chief and Associate Editors could greatly shape the process of publication in this journal. In that way this new journal can join in with several other publications that, in my view, are bringing back sanity to a process that I feel has gone largely awry in the life sciences in recent years.

First and foremost, I am convinced that we live in an era of open access and online-only publishing. This new journal adheres to these principles using the model set forth for all Nature Partner Journals. But while open access publishing is of prime importance, it is also essential to recognize that the medium is susceptible to predatory practices. Thus, there is a need to make certain that the highest scientific rigor be applied to the review process in this new journal. Here I have no doubt that it is key to have the backing of such a well-established and reputable consortium, the Nature Publishing Group.

A second strong conviction I have is that scientific publications should be both peer reviewed and peer edited. While it is wonderful that we, the editors of this new journal, will have the assistance of a great support staff, it will be practicing scientists who will be making the key decisions on submitted manuscripts. In addition, at least as far as I am concerned, I will not simply be editor for the manuscripts I handle for the journal. I will have a role of reviewing editor, providing in-depth comments along with my recommendations to the Editor-in-Chief. In the spirit of full transparency, I will also reveal my identity as reviewing editor to the authors irrespective of the decision reached on their manuscript.

For authors submitting manuscripts to journals, the review process often has the feel of a ‘black box’. Their work goes in and some time later comes out with a decision based on some comments that do not always make complete sense. My hope is that the review process in *npj Biofilms and Microbiomes* be done in a way that the authors get a clear sense of how their submission was evaluated. For every manuscript I handle, I aim to provide as a review a single consensus opinion, reached at by consultation among the reviewers and the Associate Editor. I hope this will eventually become routine for all submissions to the journal.

One key aspect that I will push for in the review process will be the elimination of what Hidde Ploegh referred to as the ‘wasteful tyranny of reviewer experiments’. If enough additional experiments are really required, that should be grounds for rejection of a manuscript. But, I will not tolerate anonymous reviewers requiring additional experiments simply because they are possible and because they would be a nice addition.

As a final note, I would like to add that I am a strong supporter of the San Francisco Declaration of Research Assessment (DORA—http://am.ascb.org/dora/). The journal impact factor has a number of well-documented deficiencies as a tool for research assessment, as such I make a commitment to attempt assessing the potential impact of individual articles based on my experience. Each article published in *npj Biofilms and Microbiomes* will also display a range of individual article metrics, allowing readers to judge each article according to its own unique impact across a broad range of criteria.

It is because there is still much work to be done in the area of scientific publishing, along with the exciting new discoveries in the areas of biofilms and microbiomes, that I have enthusiastically agreed to serve as Associate Editor of this new journal.
